# MiR-148a-3p Promotes Colorectal Cancer Cell Ferroptosis by Targeting SLC7A11

**DOI:** 10.3390/cancers15174342

**Published:** 2023-08-30

**Authors:** Elisa Martino, Anna Balestrieri, Francesca Aragona, Giovanna Bifulco, Luigi Mele, Giuseppe Campanile, Maria Luisa Balestrieri, Nunzia D’Onofrio

**Affiliations:** 1 Department of Precision Medicine, University of Campania Luigi Vanvitelli, Via L. De Crecchio 7, 80138 Naples, Italy; elisa.martino@unicampania.it (E.M.); nunzia.donofrio@unicampania.it (N.D.); 2 Food Safety Department, Istituto Zooprofilattico Sperimentale del Mezzogiorno, 80055 Portici, Italy; anna.balestrieri@izsmportici.it; 3 Department of Veterinary Medicine and Animal Production, University of Naples Federico II, 80137 Naples, Italy; francesca.aragona@unina.it (F.A.); giovanna.bifulco@unina.it (G.B.); giuseppe.campanile@unina.it (G.C.); 4 Department of Experimental Medicine, University of Campania Luigi Vanvitelli, Via Luciano Armanni 5, 80138 Naples, Italy; luigi.mele@unicampania.it

**Keywords:** colorectal cancer, miR-148a-3p, ferroptosis, lipid peroxidation, SLC7A11

## Abstract

**Simple Summary:**

The role of miR-148a-3p in colorectal cancer (CRC) is still debated. In this study, the in vitro antineoplastic effect of miR-148a-3p overexpression in the CRC model is reported. The antitumor activity of miR-148a-3p occurs through apoptosis, mitochondrial impairment, lipid peroxidation, and ferroptosis sustained by the ACSL4/TFRC/Ferritin axis. Bioinformatic analysis and transfection experiments with miR-148a-3p mimics and inhibitors revealed that the cytotoxicity might be related to the downregulation of SLC7A11. These findings, extending knowledge on functional and molecular mechanisms, unveil the oncosuppressor role of miR-148a-3p, pointing out its potential as a diagnostic and therapeutic biomarker in CRC.

**Abstract:**

Ferroptosis, an iron-dependent form of cell death, and dysregulated microRNA (miRNA) expression correlate with colorectal cancer (CRC) development and progression. The tumor suppressor ability of miR-148a-3p has been reported for several cancers. Nevertheless, the role of miR-148a-3p in CRC remains largely undetermined. Here, we aim at investigating the molecular mechanisms and regulatory targets of miR-148a-3p in the CRC cell death mechanism(s). To this end, miR-148a-3p expression was evaluated in SW480 and SW620 cells and normal colon epithelial CCD 841 CoN cells with quantitative real-time polymerase chain reaction (qRT-PCR). Data reported a reduction of miR-148a-3p expression in SW480 and SW620 cells compared to non-tumor cells (*p* < 0.05). Overexpression of miR-148a selectively inhibited CRC cell viability (*p* < 0.001), while weakly affecting normal CCD 841 CoN cell survival (*p* < 0.05). At the cellular level, miR-148a-3p mimics promoted apoptotic cell death via caspase-3 activation (*p* < 0.001), accumulation of mitochondrial reactive oxygen species (ROS) (*p* < 0.001), and membrane depolarization (*p* < 0.001). Moreover, miR-148a-3p overexpression induced lipid peroxidation (*p* < 0.01), GPX4 downregulation (*p* < 0.01), and ferroptosis (*p* < 0.01), as revealed by intracellular and mitochondrial iron accumulation and ACSL4/TFRC/Ferritin modulation. In addition, levels of SLC7A11 mRNA and protein, the cellular targets of miR-148a-3p predicted by bioinformatic tools, were suppressed by miR-148a-3p’s overexpression. On the contrary, the downregulation of miR-148a-3p boosted SLC7A11 gene expression and suppressed ferroptosis. Together, these in vitro findings reveal that miR-148a-3p can function as a tumor suppressor in CRC by targeting SLC7A11 and activating ferroptosis, opening new perspectives for the rationale of therapeutic strategies through targeting the miR-148a-3p/SLC7A11 pathway.

## 1. Introduction

The incidence of colorectal cancer (CRC) in 2020 still represented 10% of the total cancer cases and was the second cause of tumor-dependent mortality in the world, with 3.2 million new cases expected by 2040 [1,2]. Despite the rapid development of early screening methods and improved therapies, the prognosis for CRC is still not satisfactory. Among the different tools suitable for tumor screening, microRNAs (miRNAs) represent crucial biomarkers in CRC patients [3].

MiRNAs, as small non-coding RNA sequences, modify gene expression through complementary base pairing with target mRNAs, affecting the integrity or translation efficiency of approximately 60% of the human genes [4]. The involvement of miRNAs in the development and progression of several tumors has been widely reported, given their ability to regulate multiple crucial cellular functions such as proliferation, cell cycle, apoptosis, and drug response [5,6,7]. Increasing evidence reveals that non-coding RNA overexpression or depletion accelerates CRC development and growth, representing potential molecular prognostic and therapeutic biomarkers [8]. Indeed, aberrant expression of several miRNAs, including miR-21, miR-146b, miR-342-3p, miR-185-5p, miR-675-5p, miR-128-1-5p, miR-138-5p, and miR-451, is frequently found in CRC [9,10,11,12,13,14,15,16]. Moreover, the miR-148a-3p gene, located on the 7p15.2 region of human chromosomes, also plays a critical role in the carcinogenesis and progression of many cancer types, including CRC [17,18,19,20]. MiR-148a-3p belongs to the miR-148/152 family, which includes miR-148a, miR-148b, and miR-152 [21]. Contrasting evidence reported the association of miR-148a with epithelial-mesenchymal transition and a more aggressive CRC phenotype, as well as its overexpression inhibiting CRC proliferation and migration, reducing tumorigenesis, and causing early recurrence [22,23]. Therefore, the exact role of miR-148a in CRC remains largely undefined.

Ferroptosis is a novel form of programmed cell death featuring iron overload, which, via depletion of reduced glutathione (GSH) and/or the inhibition of glutathione peroxidase 4 (GPX4), enhances intracellular reactive oxygen species (ROS) and lipid peroxidation, causing membrane impairment and severe cell damage [24,25]. Inactivation of GPX4, responsible for biomembrane preservation from peroxidation damage, leads to an accumulation of lipid peroxide species [26,27]. To date, ferroptosis has emerged as a considerable strategy in the treatment of many cancers, although the malignancies susceptible to ferroptosis have not been clearly defined [28]. However, recent studies reveal an association between ferroptosis and CRC carcinogenesis [29,30]. The induction of ferroptosis by intracellular Fe^2+^ and ROS accumulation, along with reduced GPX4 and GSH content in CRC cells, contribute to the clinical treatment of this neoplasia [31,32]. On the contrary, inhibition of ferroptosis may lead to CRC progression and drug resistance [33]. More recently, the induction of ferroptosis has been described as a consequence of the inhibition of solute carrier family seven-member 11 (SLC7A11), a multichannel transmembrane protein [34,35,36], resulting in a potential antineoplastic therapeutic approach in different tumors. Of interest, SLC7A11 expression is positively associated with microsatellite instability, a phenomenon of molecular fingerprinting related to CRC prognosis [37,38]. MiR-148a-3p directly targeted SLC7A11 and inhibited its expression. Based on these results, the miR-148a-3p/SLC7A11 axis might be a novel therapeutic target for colon cancer. Although miR-148a has been reported to be associated with a good prognosis for patients with CRC and its expression is associated with tumor metastasis, the specific mechanism of action remains unclear. In this study, we aimed at investigating the biological functions of miR-148a-3p in SW480 and SW620 CRC cells and the possible role of the miR-148a-3p/SLC7A11 axis in the definition of a novel potential molecular candidate for CRC therapies.

## 2. Materials and Methods

### 2.1. Cell Culture and Transfection

Human colon epithelial CCD 841 CoN (CRL-1790, ATCC, Manassas, VA, USA) and colorectal adenocarcinoma SW480 (CCL-228, ATCC, Manassas, VA, USA) and SW620 (CCL-227, ATCC, Manassas, VA, USA) cells were grown as previously reported [39]. Cells were transfected for 6 h in serum- and antibiotic-free medium with 40 nM miRNA mimic Negative Control (miR-NC, MCH00000), hsa-miR-148a miRNA mimic (miR-148a^+^, MCH01336), hsa-miR-148a miRNA agomir (a-miR-148a, MAH01336), or miRNA agomir Negative Control (a-miR-NC, MAH00000), all from Applied Biological Materials, Inc., Richmond, BC, Canada, before the addition of fetal bovine serum (FBS). Lullaby (LL70500, OZ Biosciences, Marseille, France) was used for transfection following the manufacturer’s instructions. The cells were incubated up to 72 h after transfection.

### 2.2. Quantitative Real-Time PCR (qRT-PCR)

The evaluation of hsa-miR-148a-3p expression and SLC7A11 mRNA levels was performed as previously reported [8,40]. ID3EAL Individual miRNA RT Primer 1-plex (1103111-HSA0000243A, MiRXES, Singapore) and ID3EAL miRNA qPCR (1104101-HSA0000243A, MiRXES, Singapore) primers were used to detect hsa-miR-148a-3p levels, while the assessment of SLC7A11 mRNA was performed with the following primers: F: 5′-GCTGTGATATCCCTGGCATT-3′, R: 5′-GGCGTCTTTAAAGTTCTGCG-3′ and normalized against GAPDH [40]. The relative amount was determined using the 2^−ΔΔCt^ method [8,40].

### 2.3. Viability and Cytotoxicity

Cell viability and cytotoxicity were evaluated by cell counting kit-8 and Cytotoxicity LDH assays (CK04 and CK12, both from Donjindo Molecular Technologies, Inc., Rockville, MD, USA), respectively, following the manufacturer’s instructions. Absorbances (450 and 490 nm) were detected by a microplate reader (model 680, Bio-Rad, Hercules, CA, USA). Experiments were conducted with *n* = 4 replicates, and results were reported as %.

### 2.4. Apoptotic Cell Death

The analysis of the apoptotic mechanism was performed by the FITC annexin V apoptosis detection kit (556547, BD Pharmigen, Franklin Lakes, NJ, USA), while the caspase-3/7 detection was assessed by the Nucview 488 caspase-3 substrate and annexin V kit (30067, Biotium, Fremont, CA, USA), as already described [39,40]. For both assays, fluorescence intensities were recorded by a BD Accuri C6 cytometer (BD Biosciences, San José, CA, USA), collecting at least 20,000 events for each sample, and the data were analyzed by FlowJo V10 software (Williamson Way, Ashland, OR, USA).

### 2.5. Mitochondrial State

Mitochondrial integrity and ROS levels were assessed by staining cells for 20 min with 5 µM MitoTracker Green FM (M7514, Invitrogen, Waltham, MA, USA) and MitoSOX Red Mitochondrial Superoxide Indicator (M36008, Invitrogen, Waltham, MA, USA) fluorescent probes. The JC-1 stain (MT09, Donjindo Molecular Technologies, Inc., Rockville, MD, USA) was used to measure mitochondrial potential membranes, as previously reported [41]. The detection of ferrous ions (Fe^2+^) in mitochondria was carried out with the Mito-FerroGreen (M489; Donjindo Molecular Technologies, Inc., Rockville, MD, USA) fluorescent probe, following the supplier’s instructions. Briefly, after transfection, cells were stained with a 5 μM working solution and incubated for 20 min at 37 °C. After each staining procedure, fluorescent images were collected by an EVOS M5000 microscope (Thermo Scientific, Rockford, IL, USA), while fluorescent signals were recorded by a BD Accuri C6 cytometer (BD Biosciences, San José, CA, USA). Data analysis was performed by FlowJo V10 software (Williamson Way, Ashland, OR, USA).

### 2.6. Lipid Peroxidation 

Lipid peroxidation assay (ab118970, Abcam, Cambridge, UK) and GSSG/GSH quantification kit (G257, Dojindo Molecular Technologies, Inc., Rockville, MD, USA) were used to detect MDA content and GSH/GSSG ratio, respectively, as already reported [40]. Liperfluo (L248, Dojindo Molecular Technologies, Inc., Rockville, MD, USA) was used to evaluate lipid peroxide content according to the manufacturer’s protocols. After transfection, staining was carried out with 1 μM Liperfluo in serum-free medium for 30 min at 37 °C. Cells were imaged by an EVOS M5000 microscope (Thermo Scientific, Rockford, IL, USA), fluorescence was recorded by a BD Accuri C6 cytometer (BD Biosciences, San José, CA, USA), and results were analyzed by FlowJo V10 software (Williamson Way, Ashland, OR, USA).

### 2.7. Ferroptotic Mechanism 

A cell ferrous iron colorimetric assay (E-BC-K881-M, Elabscience Biotechnology Inc., Houston, TX, USA) was performed to measure the intracellular Fe^2+^ content, following the manufacturer’s instructions. After transfection, 1 × 10^6^ cells were centrifuged for 10 min at 1500× *g* at 4 °C and then incubated with 80 μL of reagent for 10 min at 37 °C. The 593 nm absorbance was measured with the microplate reader (model 680, Bio-Rad, Hercules, CA, USA), and the Fe^2+^ concentration was derived from the cell ferrous iron standard curve. Ferroptosis was investigated by using the fluorescent probe FerroOrange (F374, Dojindo Molecular Technologies, Inc., Rockville, MD, USA), according to the supplier’s guidance. Cells were incubated for 20 min at 37 °C with 1 μM FerroOrange working solution before fluorescent image acquisition by an EVOS M5000 microscope (Thermo Scientific, Rockford, IL, USA). Fluorescence intensity was recorded by a BD Accuri C6 cytometer (BD Biosciences, San José, CA, USA), and analysis was carried out by FlowJo V10 software (WilliamsonWay, Ashland, OR, USA).

### 2.8. Cell lysis, Immunoblotting, and Antibodies

Protein lysis and separation, nitrocellulose membrane transfer, chemiluminescent acquisition, and analysis were performed as already described [41,42], with Bcl-2 (1:500, E-AB-15522, Elabscience Biotechnology Inc., Houston, TX, USA), Glutathione Peroxidase 4 (GPX4, 1:500, ab231174, Abcam, Cambridge, UK), Acyl CoA synthetase long chain family member 4 (ACSL4, 1:1000, PA5-27137, Invitrogen, Waltham, MA, USA), Ferritin (1:1000, MA5-32244, Invitrogen, Waltham, MA, USA), xCT/SLC7A11 (1:1000, 12691, Cell Signaling Technology, Danvers, MA, USA), CD71/Transferrin Receptor (TFRC, 1:1000, sc-65882, Santa Cruz Biotechnology, Dallas, TX, USA), α-tubulin (1:5000, E-AB-20036, Elabscience Biotechnology Inc., Houston, TX, USA), actin (1:3000, ab179467, Abcam, Cambridge, UK), and GAPDH (1:2000, ab9485, Abcam, Cambridge, UK) as primary antibodies.

### 2.9. Bioinformatics Analysis

Predicted target genes for miR-148a-3p and the complementarity site in the 3′UTR of SLC7A11 mRNA were forecasted by different bioinformatic tools, such as miRDB, mirDIP, and TargetScan. The MiRDB database (http://www.mirdb.org/cgi-bin/target_detail.cgi?targetID=598538, accessed on 25 August 2023) identified SLC7A11 3′ UTR region as an hsa-miR-148a-3p target with 56 as a score value, as well as the mirDIP engine (https://ophid.utoronto.ca/mirDIP/index.jsp#r, accessed on 25 August 2023) confirmed SLC7A11 as an hsa-miR-148a-3p target with the top 5% (high) score class and 0.77 as an integrated score value. Bioinformatics analysis by using TargetScan 8.0 database (https://www.targetscan.org/cgi-bin/targetscan/vert_80/targetscan.cgi?species=Human&gid=&mir_sc=&mir_c=&mir_nc=&mir_vnc=&mirg=hsa-miR-148a-3p, accessed on 25 August 2023) assigned 66 as context score percentile and −0.10 as context score value to the conserved position 6673–6679 of SLC7A11 3′ UTR as hsa-miR-148a-3p binding site.

### 2.10. Statistical Analysis

GraphPad Prism software version 9.1.2 (GraphPad Software Inc., La Jolla, CA, USA) was used to analyze the data. Results were presented as mean ± standard deviation (SD). Using test t or ANOVA and Tukey’s, the significance was examined and defined as statistically significant as a *p* < 0.05.

## 3. Results

### 3.1. MiR-148a Levels in CRC

**Figure 1:**
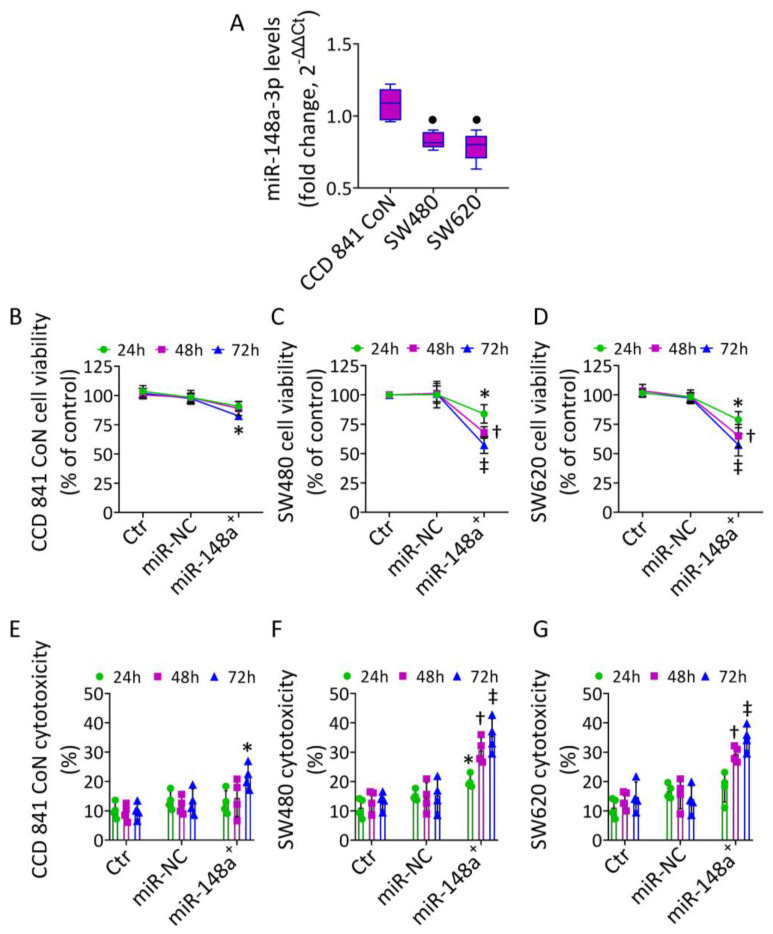
MiR-148a^+^-mediated cytotoxicity in CRC. (**A**) Basal hsa-miR-148a-3p expression, measured by qRT-PCR, in CCD 841 CoN, SW480, and SW620 cells reported as floating bars with the line representing the mean ± SD. Cell viability and cytotoxicity evaluated up to 72 h after transfection with miRNA mimic Negative Control (miR-NC) or hsa-miR-148a miRNA mimic (miR-148a^+^) on (**B**,**E**) CCD 841 CoN, (**C**,**F**) SW480, and (**D**,**G**) SW620 cells. The data are expressed as the mean ± SD of *n* = 3 experiments. • *p* < 0.05 vs. CCD 841 CoN cells; * *p* < 0.05 vs. miR-NC; † *p* < 0.01 vs. miR-NC; ‡ *p* < 0.001 vs. miR-NC.

The expression of miR-148a-3p was determined in normal colon CCD 841 CoN and colorectal adenocarcinoma SW480 and SW620 cells (Figure 1). The qRT-PCR analysis revealed that the expression level of miR-148a-3p was considerably lower in SW480 (0.83 ± 0.06-fold change vs. 1.08 ± 0.11-fold) and SW620 cells (0.79 ± 0.10-fold change vs. 1.08 ± 0.11-fold change) compared to normal CCD 841 CoN cells (*p* < 0.05) (Figure 1A).

To study the biological role of miR-148a-3p in CRC, the different cell lines were transfected with miR-148a mimic (miR-148a^+^) (Figure S1). Overexpression of miR-148a notably decreased SW480 and SW620 cell viability, with the highest effects at 72 h after transfection (*p* < 0.001 vs. miR-NC) (Figure 1C,D). This effect was accompanied by increased cytotoxicity (*p* < 0.001 vs. miR-NC) (Figure 1F,G). On the contrary, miR-148a overexpression affected the viability and cytotoxicity of CCD 841 CoN to a lesser extent (*p* < 0.05 vs. miR-NC) (Figure 1B,E).

### 3.2. MiR-148a^+^ Induced Caspase-3-Dependent Apoptosis in CRC

**Figure 2:**
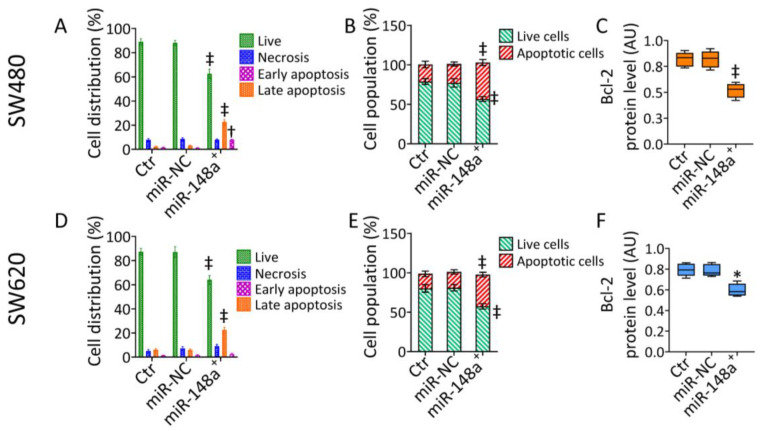
MiR-148a^+^-related apoptosis. Representative (**A**,**D**) annexin V-FITC and PI-staining and (**B**,**E**) caspase-3 activation, detected by FACS analysis, and (**C**,**F**) immunoblotting evaluation of Bcl-2 protein levels in SW480 and SW620 cells. Western blotting results are reported as arbitrary units (AU). * *p* < 0.05 vs. miR-NC; † *p* < 0.01 vs. miR-NC; ‡ *p* < 0.001 vs. miR-NC.

To investigate the miR-148a-mediated cytotoxicity, apoptotic cell death was then explored (Figure 2 and Figure S2).

The results indicated that miR-148a^+^ decreased live cells (65.55% ± 3.83 vs. 88.00% ± 2.13 in miR-NC, *p* < 0.001) and induced early (7.96% ± 0.38 vs. 1.21% ± 0.35 in miR-NC, *p* < 0.01) and late apoptotic accumulation (22.69% ± 2.04 vs. 3.01% ± 0.46 in miR-NC, *p* < 0.001) in SW480 cells (Figure 2A and Figure S2). The miR-148a-induced apoptosis was associated with caspase-3 activation (*p* < 0.001 vs. miR-NC) (Figure 2B and Figure S2) and a notable depletion of mitochondrial Bcl-2 protein levels (*p* < 0.001 vs. miR-NC) (Figure 2C and Figure S2). Similarly, in SW620 cells, miR-148a^+^ reduced the live cell population (64.05% ± 3.64 vs. 87.08% ± 4.44 in miR-NC, *p* < 0.001) while increasing the late apoptotic rate (22.45% ± 2.28 vs. 5.90% ± 1.02 in miR-NC, *p* < 0.001) (Figure 2D and Figure S2) via caspase-3 activation (*p* < 0.001 vs. miR-NC) (Figure 2E and Figure S2). The apoptotic mechanism resulted in the downregulated expression of Bcl-2 (*p* < 0.05 vs. miR-NC) (Figure 2F and Figure S2).

### 3.3. MiR-148a^+^ Triggered Mitochondrial Damage in CRC

**Figure 3:**
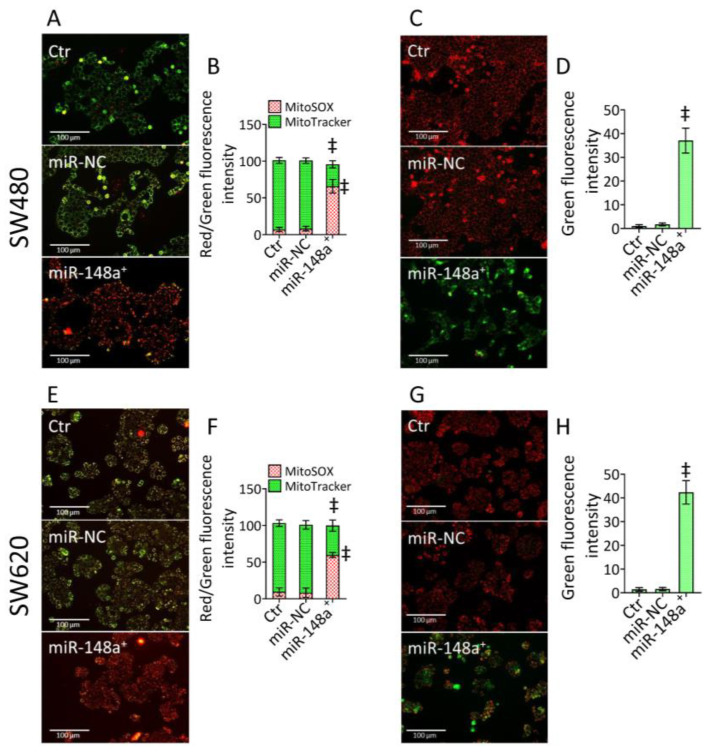
MiR-148a^+^-related mitochondrial impairment. Representative fluorescent images and cytofluorimetric evaluation of (**A**,**B**,**E**,**F**) mitochondrial integrity (green) and ROS content (red) and (**C**,**D**,**G**,**H**) mitochondrial membrane potential in SW480 and SW620 cells. Scale bars = 100 μm. The data are expressed as the mean ± SD of *n* = 3 experiments. ‡ *p* < 0.001 vs. miR-NC.

Mitochondrial impairment was analyzed using different approaches. Firstly, double staining with MitoTracker and MitoSOX fluorescent probes was used to evaluate mitochondrial integrity and the occurrence of mitochondrial oxidative stress, respectively (Figure 3 and Figure S3).

Results revealed that miR-148a^+^ promoted mitochondrial ROS accumulation and decreased mitochondrial integrity (*p* < 0.001 vs. miR-NC) (Figure 3 and Figure S3). Fluorecence and cytometric analysis of mitochondrial membrane potential showed the miR-148a^+^ capacity to provoke an impressive membrane depolarization rate (*p* < 0.001 vs. miR-NC) (Figure 3 and Figure S3).

### 3.4. MiR-148a^+^ Promoted Lipid Peroxidation in CRC

**Figure 4:**
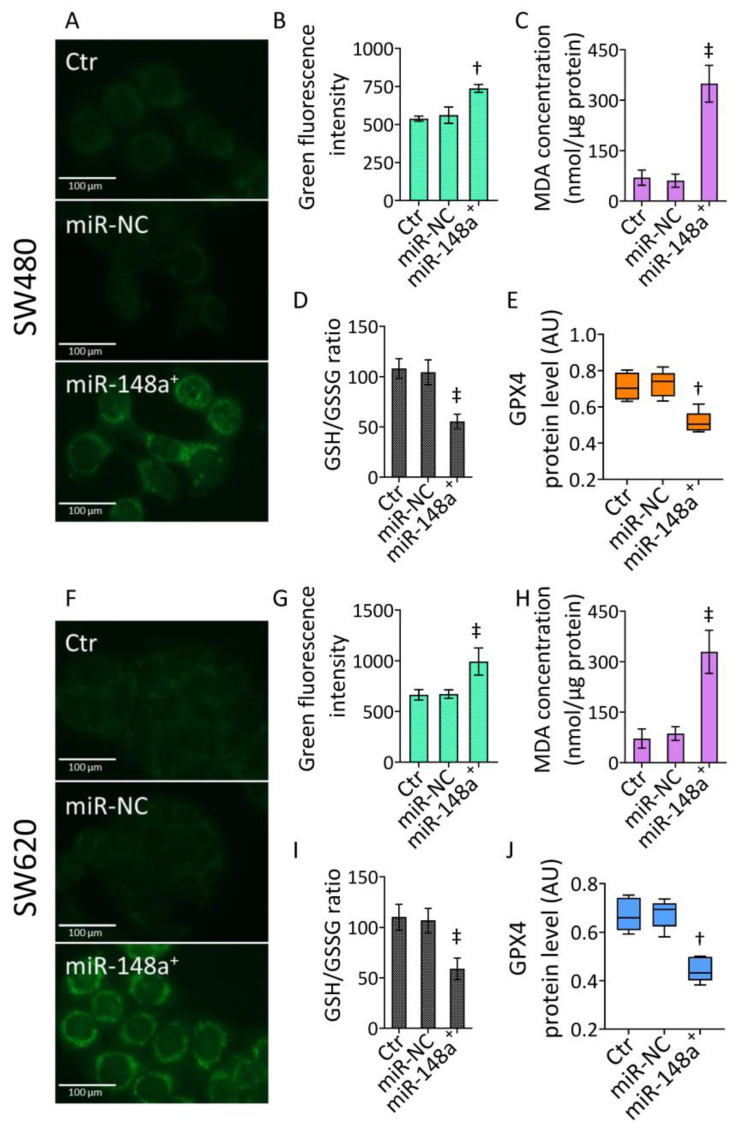
MiR-148a^+^-derived lipid peroxidation. (**A**,**B**,**F**,**G**) Representative fluorescent images and FACS analysis of lipid peroxide levels, evaluation of (**C**,**H**) MDA and (**D**,**I**) GSH/GSSG content, and (**E**,**J**) western blotting detection of GPX4 protein levels in SW480 and SW620 cells. Scale bars = 100 μm. The data are expressed as the mean ± SD of *n* = 3 experiments. Western blotting results are reported as arbitrary units (AU). † *p* < 0.01 vs. miR-NC; ‡ *p* < 0.001 vs. miR-NC.

The accumulation of oxidative damage to mitochondria led to the exploration of the occurrence of lipid peroxidation (Figure 4 and Figure S4), closely involved in mitochondrial detriment.

The Liperfluo fluorescent probe detected lipid peroxide (*p* < 0.01 vs. miR-NC), MDA accumulation (*p* < 0.001 vs. miR-NC), and impaired GSH/GSSG ratio (*p* < 0.001 vs. miR-NC) in SW480 cells transfected with miR-148a^+^ (Figure 4A–D and Figure S4). These effects were accompanied by downregulated GPX4 protein levels (*p* < 0.01 vs. miR-NC) (Figure 4E and Figure S4). Likewise, miR-148a^+^ triggered lipid peroxidation, MDA accrual, and GSH/GSSG decrease (*p* < 0.001 vs. miR-NC), as well as reduced GPX4 expression (*p* < 0.01 vs. miR-NC) in SW620 cells (Figure 4F–J and Figure S4).

### 3.5. MiR-148a^+^ Provoked Ferroptosis in CRC

**Figure 5:**
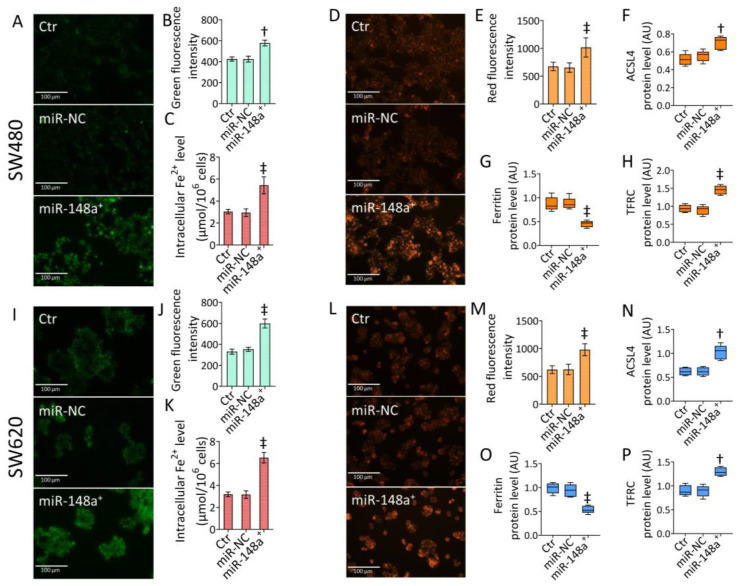
MiR-148a^+^-sustained ferroptosis. Representative fluorescent images and cytofluorimetric evaluation of (**A**,**B**,**I**,**J**) mitochondrial Fe^2+^ content and (**D**,**E**,**L**,**M**) ferroptosis, (**C**,**K**) assessment of intracellular Fe^2+^ levels, and western blotting detection of (**F**,**N**) ACSL4, (**G**,**O**) ferritin, and (**H**,**P**) TFRC protein levels in SW480 and SW620 cells. Scale bars = 100 μm. The data are expressed as the mean ± SD of *n* = 3 experiments. Western blotting results are reported as arbitrary units (AU). † *p* < 0.01 vs. miR-NC; ‡ *p* < 0.001 vs. miR-NC.

Mitochondrial oxidative imbalance and lipid peroxidation are critical events in ferroptosis. Therefore, iron-dependent cell death was investigated (Figure 5 and Figure S5).

In SW480 cells, miR-148a overexpression induced accumulation of intracellular (*p* < 0.001 vs. miR-NC) and mitochondrial ferrous (Fe^2+^) ions (*p* < 0.01 vs. miR-NC) (Figure 5A–C and Figure S5). The miR-148a-dependent iron accrual resulted in ferroptosis induction (*p* < 0.001 vs. miR-NC), as assessed by fluorescence and cytofluorimetric analysis (Figure 5D,E and Figure S5), sustained by increased ACSL4 and TFRC expression and reduced ferritin protein levels (*p* < 0.001 vs. miR-NC) (Figure 5F–H and Figure S5). Similar results were obtained in SW620 cells. In detail, overexpression of miR-148a increased intracellular and mitochondrial iron content (*p* < 0.001 vs. miR-NC), resulting in ferroptosis induction (*p* < 0.001 vs. miR-NC) (Figure 5I–M and Figure S5). SW620 cells transfected with miR-148a^+^ showed upregulated ACSL4 and TFRC protein levels (*p* < 0.01 vs. miR-NC) and depleted ferritin expression (*p* < 0.001 vs. miR-NC) (Figure 5N–P and Figure S5).

### 3.6. SLC7A11 as a Target of miR-148a

**Figure 6:**
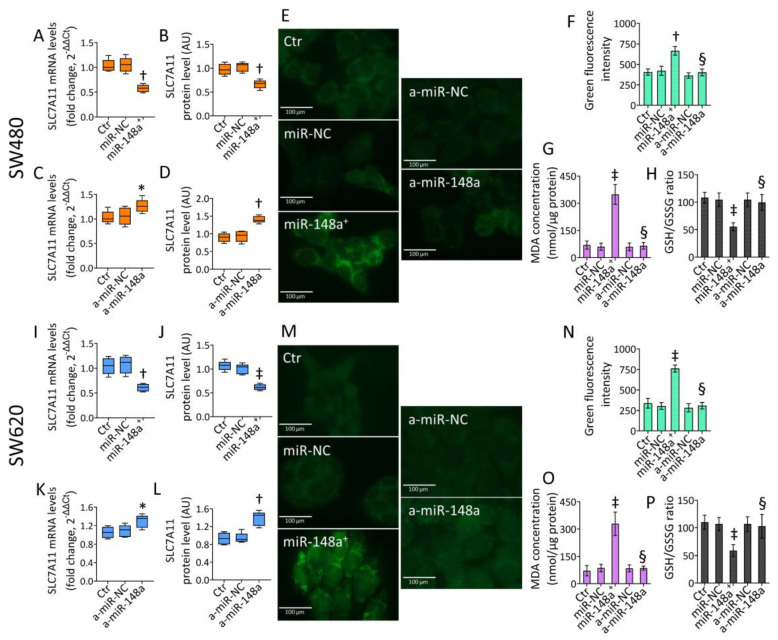
SLC7A11 as a miR-148a target. Representative qRT-PCR and western blotting detection of SLC7A11 mRNA and protein levels evaluated in SW480 and SW620 cells transfected with (**A**,**B**,**I**,**J**) miRNA mimic Negative Control (miR-NC) or hsa-miR-148a miRNA mimic (miR-148a^+^) or with (**C**,**D**,**K**,**L**) miRNA agomir Negative Control (a-miR-NC) or hsa-miR-148a miRNA agomir (a-miR-148a). Western blotting results are reported as arbitrary units (AU). (**E**,**F**,**M**,**N**) Representative fluorescent images and cytofluorimetric detection of lipid peroxide content, and evaluation of (**G**,**O**) MDA and (**H**,**P**) GSH/GSSG levels in SW480 and SW620 cells. Scale bars = 100 μm. The data are expressed as the mean ± SD of *n* = 3 experiments. * *p* < 0.05 vs. a-miR-NC; † *p* < 0.01 vs. miR-NC or a-miR-NC; ‡ *p* < 0.001 vs. miR-NC; § *p* < 0.01 vs. miR-148a^+^.

Bioinformatics analyses predicted SLC7A11 as a target of miR-148a-3p. Therefore, the effects of miR-148a^+^ and agomir-148a (a-miR-148a) on SLC7A11 expression were evaluated (Figure 6 and Figure S6). In accordance with the bioinformatic prediction, miR-148a^+^ reduced both SLC7A11 mRNA and protein levels in SW480 and SW620 cells (*p* < 0.01 vs. miR-NC) (Figure 6A,B,I,J and Figure S6).

To further confirm SLC7A11 as a miR-148a target, CRC cells were transfected with a-miR-148a, and then SLC7A11 expression was evaluated (Figure 6 and Figure S6). Results showed a marked upregulation of SLC7A11 mRNA and protein levels in a-miR-148a-transfected cells (*p* < 0.01 vs. a-miR-NC) (Figure 6C,D,K,L and Figure S6), validating the miR-148a capacity to modulate SLC7A11 expression in both CRC lines. Further functional experiments were performed with a-miR-148a to corroborate the miR-148a-mediated ferroptosis as a cell death mechanism and its oncosuppressor role in the in vitro CRC model.

### 3.7. miR-148a Inhibition Denied Lipid Peroxidation in CRC

Transfection with a-miR-148a strongly opposed the miR-148a^+^ ability to induce lipid peroxidation as well as MDA accumulation and GSH/GSSG decrease (*p* < 0.01 vs. miR-148a^+^) in SW480 cells (Figure 6E–H and Figure S6). Similarly, a-miR-148a denied lipid peroxide, MDA accrual, and GSH/GSSG reduction compared to miR-148a overexpression (*p* < 0.01) in the metastatic SW620 cell line (Figure 6M–P and Figure S6).

### 3.8. miR-148a Depletion Deleted Ferroptosis in CRC

**Figure 7:**
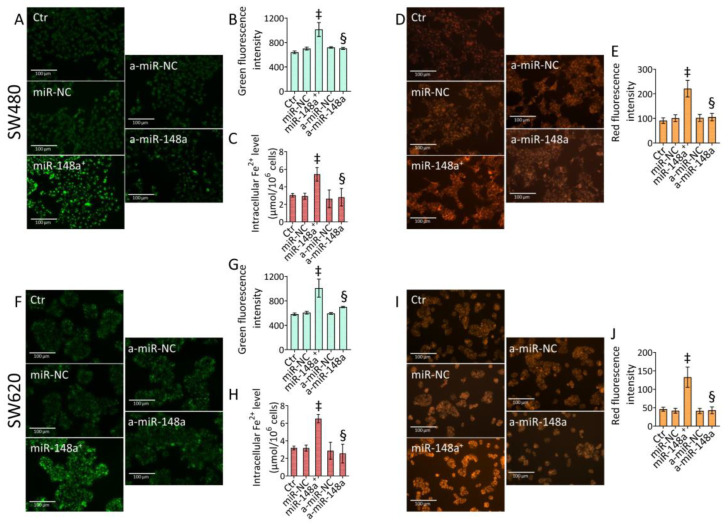
MiR-148a inhibition suppressed ferroptosis. Representative fluorescent images and cytofluorimetric detection of (**A**,**B**,**F**,**G**) mitochondrial Fe^2+^ content, (**D**,**E**,**I**,**J**) ferroptosis, and (**C**,**H**) intracellular Fe^2+^ levels evaluated in SW480 and SW620 cells. Scale bars = 100 μm. The data are expressed as the mean ± SD of *n* = 3 experiments. ‡ *p* < 0.001 vs. miR-NC; § *p* < 0.01 vs. miR-148a^+^.

Depletion of miR-148a inhibited iron accumulation and ferroptosis triggered by miR-148a^+^ (Figure 7 and Figure S7).

In SW480 cells, a-miR-148a downregulated mitochondrial and intracellular iron accumulation and ferroptotic cell death (*p* < 0.01 vs. miR-148a^+^) (Figure 7A–E and Figure S7). Moreover, in the SW620 cell line, inhibition of miR-148a opposed the miR-148a^+^ capacity to trigger ferrous ion accrual, resulting in a ferroptotic mechanism (*p* < 0.01 vs. miR-148a^+^) (Figure 7F–J and Figure S7).

## 4. Discussion

In this study, we provide the first evidence on the antiproliferative role of miR-148a-3p, showing that the overexpression of miR-148a-3p by mimic transfection exerts antitumor effects and mediates lipid peroxidation and ferroptotic cell death via the ACSL4/TFRC/Ferritin axis by targeting SLC7A11 in CRC cells.

CRC still represents one of the main causes of massive cancer-related deaths, and its invasion and metastasis significantly affect the prognosis of CRC patients [1,2]. Further studies on the underlying mechanisms of CRC initiation and progression are required to reduce mortality caused by this neoplasia. Undoubtedly, abnormal expression of miRNAs correlates with CRC pathological stage and prognosis [43,44,45,46].

MiR-148a-3p has been described as participating in various biological processes in human cancer. Several reports indicated the miR-148a-3p ability to repress proliferation and invasion in esophageal and bladder cancer, inhibit progression of epithelial ovarian cancer through directly inhibiting the expression of c-Met, as well as predict patient drug response and inhibit breast cancer progression [47,48,49,50,51]. The role of miR-148a-3p in CRC is still debated since contrasting data reported that both elevated and downregulated miR-148a-3p levels were detected in tissues from patients with advanced colorectal adenoma [20,52]. Here, we found decreased miR-148a-3p levels in SW480 and SW620 CRC cells compared to their non-tumor counterpart, consistent with previous data reporting miR-148a-3p expression was significantly downregulated in colon adenocarcinoma, thus contributing to elucidating its suppression as a crucial factor in tumor progression and poor survival in CRC [53,54]. We report that miR-148a-3p overexpression induced selective CRC cytotoxicity, triggering caspase-3-dependent apoptotic cell death via Bcl-2 downregulation, in line with evidence showing that miR-148a-3p overexpression reduced the progression of colon adenocarcinoma [55].

The search for targeted therapy in CRC aimed at interfering with critical cell mechanisms such as cell growth and proliferation, angiogenesis, migration, and differentiation focuses on miRNA’s ability to penetrate cells and inhibit target pathway(s), preventing cancer growth and causing apoptosis [43,44,45,46]. The capacity of miR-148a-3p to induce mitochondrial injury and aberrant ROS production has been reported in different cell models [56,57,58]. The results of this study provided the first evidence that overexpression of miR-148a-3p caused mitochondrial dysfunction along with oxidative stress, resulting in mitochondrial impairment and membrane depolarization in SW480 and SW620 cells. Mechanistically, miR-148a-3p mimic induces lipid peroxidation and GPX4 decreases, resulting in ferroptosis death via the ACSL4/TFRC/Ferritin signaling pathway. MiR-148a-3p overexpression increased MDA and mitochondrial and intracellular Fe^2+^ ion accumulation in CRC cell lines, thereby promoting ferroptotic cell death. The effects of ferroptosis accompanied by elevated ROS, intracellular ferrous iron, MDA, and GSH levels, as well as the involvement of the GPX4/TFRC/Ferritin axis mediated by miRNAs, were already reported as antiproliferative strategies in CRC [59,60]. Interestingly, inhibition of miR-148a-3p by agomir transfection led to a reduction in MDA and lipid peroxidation and intracellular and mitochondrial iron accumulation, resulting in a lack of miR-148a-3p-mediated ferroptosis. Results also clarified that miR-148a-3p directly targets SLC7A11 and represses its expression in vitro, promoting ferroptosis via SLC7A11 in CRC.

SLC7A11 mediates the Cys/Glu antiporter activity in the xc-system, a non-sodium-dependent transporter protein complex that outputs intracellular Glu and inputs extracellular Cys, a precursor for GSH production [61]. Several reports have elucidated that the p53-SLC7A11 axis, along with the accrual of coenzyme ubiquinone produced by the mevalonate pathway, may be linked to the ferroptosis mechanism [62]. Similarly, SLC7A11 inhibition-derived ferroptosis has been described as an escape mechanism for tumor growth in CRC [36,63,64]. However, none of these studies explored the miR-148a-3p/ferroptosis association as an antineoplastic tool in CRC. We reported that miR-148a-3p overexpression induced mitochondrial iron accumulation, lipid peroxidation, and ferroptosis via SLC7A11 modulation, sustaining the oncosuppressor action of miR-148a-3p in CRC.

Of interest, the levels of miR-148a-3p, the most abundant exosomal miRNA in human and bovine milk, are influenced by diet [65,66], and elevated levels of exosome-derived miR-148a-3p have also been characterized in milk from Mediterranean buffalo, *Bubalus bubalis*, compared to commercial cow milk [8]. Different population studies evaluated the role of the milk-abundant miR-148a-3p in breastfed children’s immunity and described the relationship between miR-148a-3p levels and food supplementation [67,68]. Recently, a randomized controlled trial assessed in breast milk the relationship among a diet integrated with *Limosilactobacillus reuteri* and omega-3 polyunsaturated fatty acids, the levels of miRNAs related to the immune system, and the frequency of infant regulatory T cells (Treg) [67]. Results correlated colostrum miR-148a-3p expression with activated Treg cells at 24 months in breast-fed infants, elucidating the role of miR-148a-3p in infant immune function [67]. Additionally, a previous trial evaluating probiotic supplementation for mothers in the perinatal period demonstrated a 40% relative risk reduction in the incidence of atopic dermatitis (AD) in infancy at 2 years of age [68]. Human breast milk samples contained some highly expressed miRNAs, including miR-148a-3p, thus displaying a pivotal role in the prevention of AD in infancy, enhanced by probiotic ingestion [68].

The relationship between modifiable lifestyles, including diet, and the onset and prevention of CRC is constantly increasing [69]. Understanding the mechanistic relationship between miR-148a-3p nutritional modulation and susceptibility to CRC development could be important for both cumulative risk reduction and the design and implementation of future public health programs and behavioral interventions.

The present work sheds light on the mechanism behind miR-148a-3p functioning in CRC and unveils its value as a novel therapeutic target for CRC treatment. Previous studies have shown the potential of circulating miR-148a-3p as a noninvasive biomarker for tumor diagnosis and monitoring. A study performed on 137 CRC patients and 145 healthy subjects showed the important dysregulation of serum miR-148a-3p in CRC patients and indicated miR-148a-3p diagnostic ability with high sensitivity and specificity for CRC detection [70]. Here, we showed that the overexpression of miR-148a-3p, by promoting ferroptosis, could represent a considerable molecular target for CRC and suggested the importance of including miR-148a-3p in a combined miR-panel for effective discrimination of CRC from non-cancerous subjects. However, other roles of miR-148a-3p in the development and prognosis of CRC warrant further investigation.

This study has several limitations to take into account. First, the reported findings only relate to the in vitro culture model, which cannot adequately represent the complexity of factors affecting in vivo CRC homeostasis. Therefore, preclinical tumor models are imperative to confirm our results. Second, the genetic profile proper of each cell line mimicking the heterogeneity and complexity of in vivo CRC, specifically SW480 as primary colon adenocarcinoma (Dukes’ type B) and SW620 as lymph node metastasis from SW480’s primary tumor site (Dukes’ type C), could explain a selective responsiveness to miR-148a-3p action. Indeed, differences in the tumor microenvironment due to the different cell models might translate into distinct susceptibilities, leading to non-translating clinical results. Third, the search and characterization of novel targets will need to be improved to better elucidate the role of miR-148a-3p in the progression and treatment of CRC. Lastly, the encapsulation of miR-148a-3p in effective and safe nanostructured carriers will allow site-specific effects able to impair tumor cells without influencing the healthy counterpart, leading to the development of personalized medicine, a promising strategy in CRC diagnosis and treatments [71].

## 5. Conclusions

Altogether, this study sheds light on the role of miR-148a-3p in CRC development and progression. Herein, we describe the tumor suppressive ability of miR-148a-3p in CRC SW480 and SW620 cells by inducing mitochondrial stress, lipid peroxidation, mitochondrial and intracellular iron accumulation, and ferroptotic cell death by SLC7A11 modulation. Overall, our study provides novel insights into the molecular pathogenesis of CRC, showing the potential of miR-148a-3p in the setting of novel strategies for the diagnosis and therapy of CRC.

## Data Availability

The data presented in this study are available from the corresponding author upon request.

## Supplementary Materials

The following supporting information can be downloaded at: https://www.mdpi.com/article/10.3390/cancers15174342/s1, Figure S1: Expression levels of miR-148a-3p in mimic transfected cells; Figure S2: Apoptotic cell death analysis; Figure S3: Mitochondrial evaluation by cytofluorimetric analysis; Figure S4: Lipid peroxidation evaluation; Figure S5: Ferroptosis analysis and markers Figure S6: SLC7A11 and miR-148a-3p levels in agomir-transfected cells; Figure S7: Cytofluorimetric analysis of ferroptosis.
