# Supplementary material for: MiR-148a-3p Promotes Colorectal Cancer Cell Ferroptosis by Targeting SLC7A11
Source: Cancers (Basel). 2023 Aug 30;15(17):4342. doi: 10.3390/cancers15174342 (PMC10486764; doi:10.3390/cancers15174342)
Supplement: Supplementary file 1 [file cancers-15-04342-s001.zip › cancers-2593397-supplementary.pdf]

MiR-148a-3p promotes colorectal cancer cell ferroptosis by targeting SLC7A11

Elisa Martino [1], Anna Balestrieri [2], Francesca Aragona [3], Giovanna Bifulco[3], Luigi Mele [4], Giuseppe Campanile [3], Maria Luisa Balestrieri [1,*] and Nunzia D'Onofrio [1]

[1]Department of Precision Medicine, University of Campania Luigi Vanvitelli, Via L. De Crecchio 7, 80138 Naples, Italy; elisa.martino@unicampania.it (E.M.), nunzia.donofrio@unicampania.it (N.D.).

[2]Food Safety Department, Istituto Zooprofilattico Sperimentale del Mezzogiorno, 80055 Portici, Italy; anna.balestrieri@izsmportici.it (A.B.).

[3]Department of Veterinary Medicine and Animal Production, University of Naples Federico II, 80137 Naples, Italy; francesca.aragona@unina.it (F.A.), giovanna.bifulco@unina.it, (G.B.), giuseppe.campanile@unina.it (G.C.).

[4]Department of Experimental Medicine, University of Campania Luigi Vanvitelli, Via Luciano Armanni 5, 80138 Naples, Italy; luigi.mele@unicampania.it (L.M.).

*****Correspondence: marialuisa.balestrieri@unicampania.it (M.L.B.); Tel.: +39-081-566-5865.

SUPPLEMENTARY MATERIALS

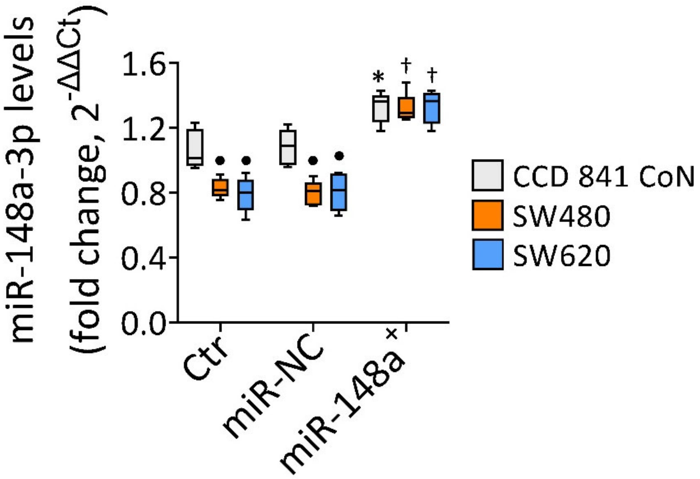

Figure S1. Expression levels of miR-148a-3p in mimic transfected cells. Hsa-miR-148a-3p expression, measured by qRT-PCR, in CCD 841 CoN, SW480 and SW620 cells transfected with miRNA mimic Negative Control (miR-NC) or hsa-miR-148a miRNA mimic (miR-148a^{+}). Data are reported as floating bars with line representing the mean ± SD of n = 3 experiments. •p<0.05 vs. CCD 841 CoN cells; *p<0.05 vs. miR-NC; †p<0.01 vs. miR-NC.

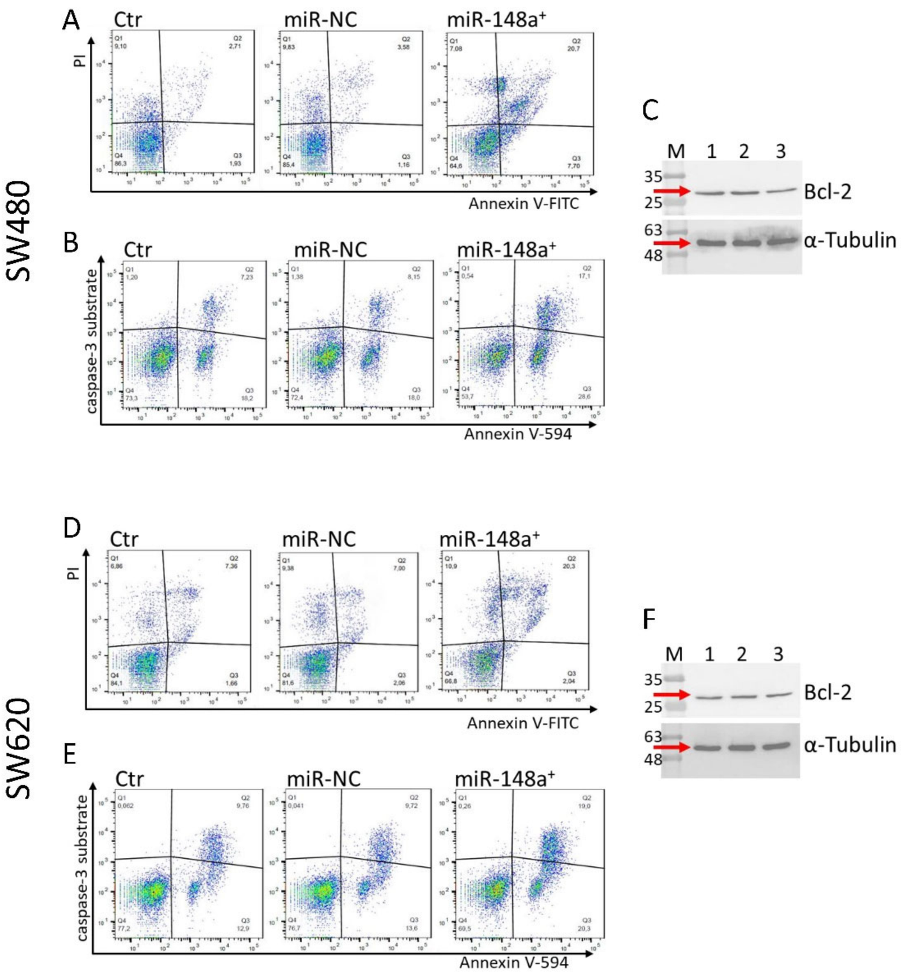

Figure S2. Apoptotic cell death analysis. Representative dot plots of (A,D) annexin V-FITC and PI-staining and (B,E) caspase-3 activation, and (C,F) cropped blots of immunoblotting analysis of Bcl-2 protein levels in SW480 and SW620 cells. Q1: necrotic cells; Q2: late apoptotic cells; Q3: early apoptotic cells; Q4: viable cells. M = molecular weight markers, lane 1 = Ctr, lane 2 = miR-NC, lane 3 = miR-148a^{+}.

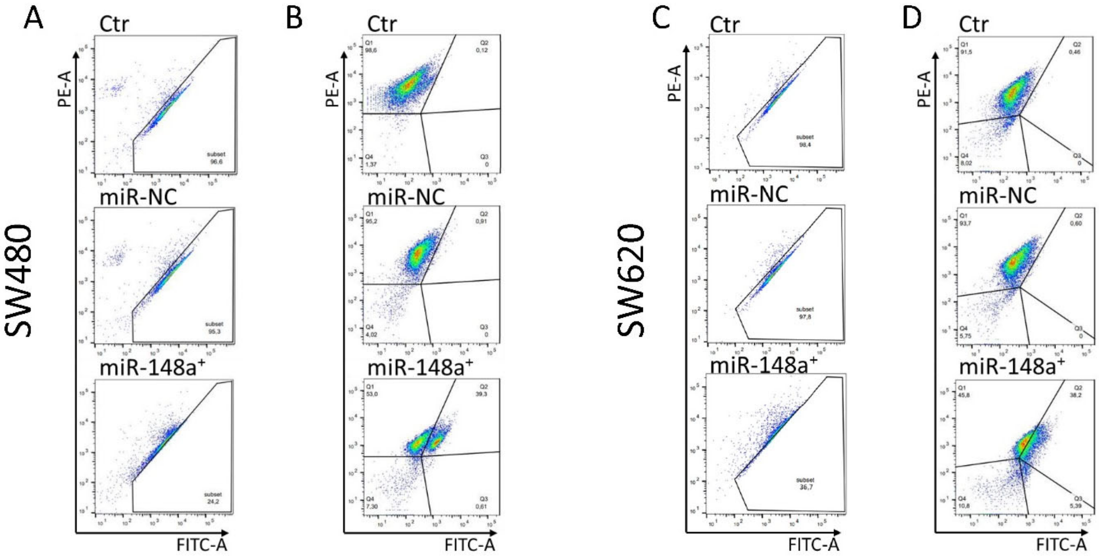

Figure S3. Mitochondrial evaluation by cytofluorimetric analysis. Representative FACS analysis of (A,C) mitochondrial integrity and (B,D) mitochondrial membrane potential in SW480 and SW620 cells.

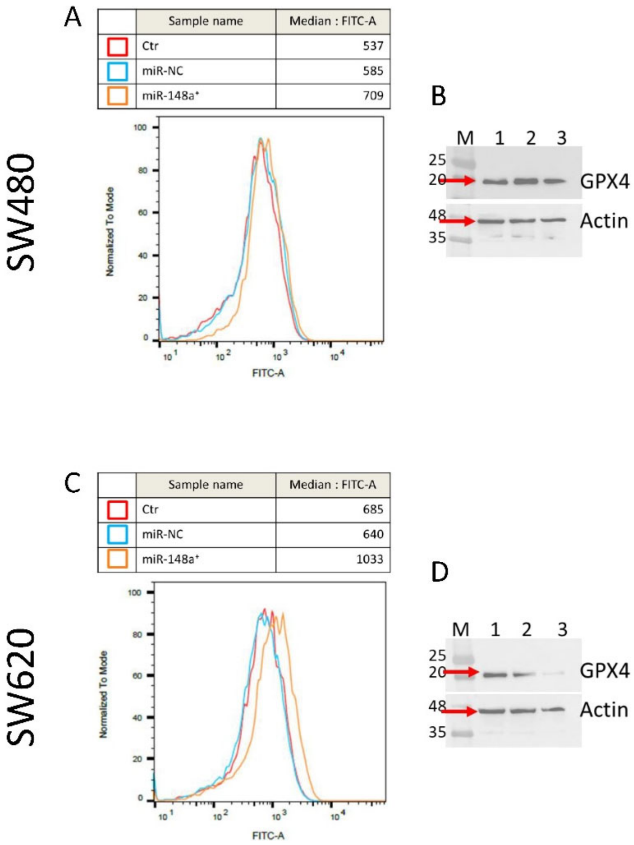

Figure S4. Lipid peroxidation evaluation. Representative FACS analysis of (A,C) lipid peroxide levels and (B,D) cropped blots of immunoblotting analysis of GPX4 protein levels in SW480 and SW620 cells. M = molecular weight markers, lane 1 = Ctr, lane 2 = miR-NC, lane 3 = miR-148a^{+}.

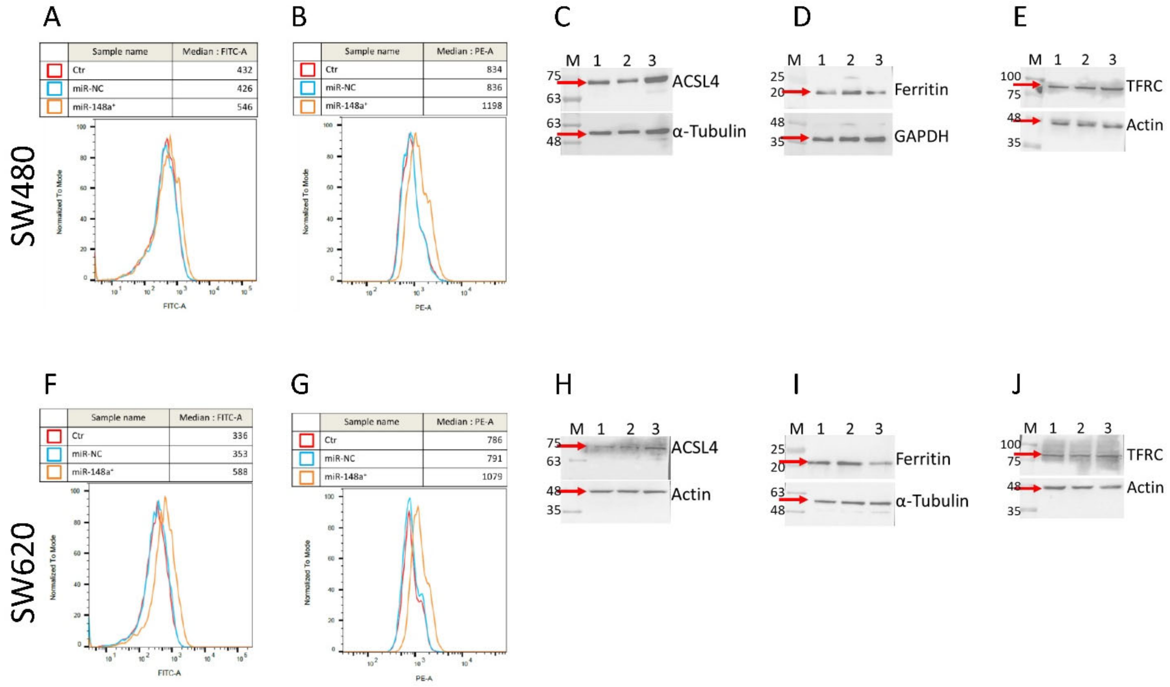

Figure S5. Ferroptosis analysis and markers. Representative FACS analysis of (A,F) mitochondrial Fe^{2+} content and (B,G) ferroptosis, and cropped blots of immunoblotting analysis of (C,H) ACSL4, (D,I) Ferritin and (E,J) TFRC protein levels in SW480 and SW620 cells. M = molecular weight markers, lane 1 = Ctr, lane 2 = miR-NC, lane 3 = miR-148a^{+}.

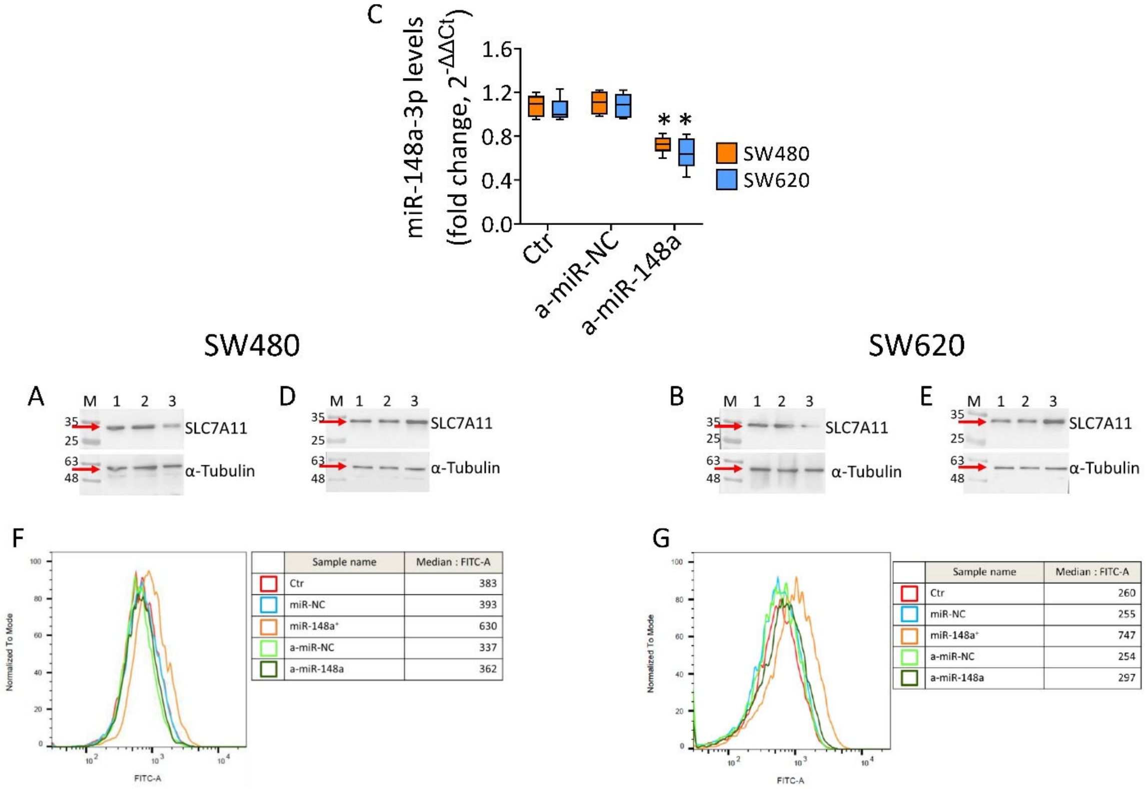

Figure S6. SLC7A11 and miR-148a-3p levels in agomir transfected cells. Representative cropped blots of immunoblotting analysis of SLC7A11 protein levels evaluated in SW480 and SW620 cells transfected with (A,B) miRNA mimic Negative Control (miR-NC) or hsa-miR-148a miRNA mimic (miR-148a^{+}) or with (D,E) miRNA agomir Negative Control (a-miR-NC) or hsa-miR-148a miRNA agomir (a-miR-148a). M = molecular weight markers, lane 1 = Ctr, lane 2 = miR-NC or a-miR-NC, lane 3 = miR-148a^{+} or a-miR-148a. (C) Hsa-miR-148a-3p expression, measured by qRT-PCR, in SW480 and SW620 cells transfected with miRNA agomir Negative Control (a-miR-NC) or hsa-miR-148a miRNA agomir (a-miR-148a). Data are reported as floating bars with line representing the mean ± SD of n = 3 experiments. *$p<0.05$ vs. a-miR-NC. (F,G) Representative FACS analysis of lipid peroxide content in SW480 and SW620 cells.

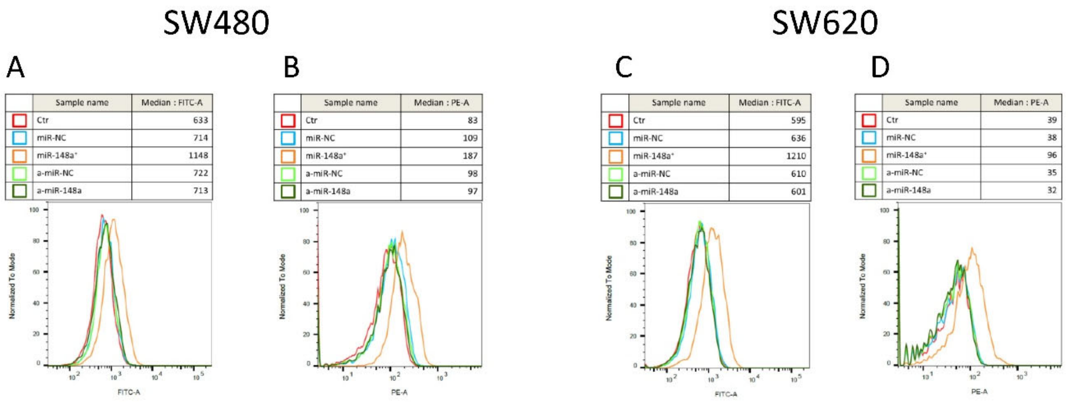

Figure S7. Cytofluorimetric analysis of ferroptosis. Representative FACS analysis of (A,C) mitochondrial Fe^{2+} content and (B,D) ferroptosis evaluated in SW480 and SW620 cells.
